# Utilizing shRNA-expressing lentivectors for viral hemorrhagic septicemia virus suppression via NV gene targeting

**DOI:** 10.3389/fvets.2025.1508470

**Published:** 2025-04-04

**Authors:** Negin Pourhoseini Dehkordi, Behnaz Saffar, Azam Mokhtari, Leila Asadi Samani, Azam Amini

**Affiliations:** ^1^Department of Genetics, Shahrekord University, Shahrekord, Iran; ^2^Department of Pathobiology, Faculty of Veterinary Medicine, Shahrekord University, Shahrekord, Iran

**Keywords:** VHSV, NV, RNAi, shRNA, lentiviral vector, TCID50

## Abstract

**Background:**

Viral hemorrhagic septicemia virus or VHSV, is a single-stranded negative-sense RNA virus that is a member of the *Rhabdoviridae* family’s genus *Novirhabdovirus*. Its major host is rainbow trout. Severe clinical symptoms and a higher mortality rate in fish populations are caused by this virus. Regretfully, there is currently no medication or vaccination available to treat it. Recently, there has been a lot of interest in developing antiviral therapies employing interfering RNA (RNAi), particularly shRNA. This study used shRNAs targeting the NV gene of VHSV to test its effectiveness in preventing VHSV proliferation in cell culture. Using the VHSV-Fil3 strain, the appropriate oligonucleotide sequence for NV gene coding was chosen for this purpose. Subsequently, shRNA molecules were designed and synthesized with the aid of shRNA design tools. The shRNAs were transfected into HEK293T cells after being cloned into the suitable vectors using the third generation of lentiviral packaging system. The CS2-2 cell line was subsequently transduced with these shRNA-expressing lentiviruses in order to challenge the VHS virus. Finally, TCID50 was employed to calculate the viral infectious titer in order to assess the effectiveness of shRNAs.

**Results:**

According to the final calculations, all shRNAs exhibited antiviral activity. When compared to the control groups, the shRNAs 1, 2, and 3 considerably lowered VHSV output in the TCID50 test (nearly 99.99, 99.99, and 99.99%, respectively, compared to cells with VHSV inoculation and nearly 99.98, 99.98, and 99.97%, respectively, compared to cells with VHSV and scrambled vector inoculation).

**Conclusion:**

Thus, it can be declared that RNA interference (RNAi) has the potential to be an exceptionally effective therapeutic option against viruses like VHSV.

## 1 Introduction

The viral hemorrhagic septicemia virus (VHSV) is one of the biggest threats and problematic factors in the aquaculture industry. This virus belongs to the *Novirhabdovirus* genus of the *Rhabdoviridae* family and is considered an important source of mortality among farmed and free-living freshwater and marine fish worldwide. This virus is epidemic in most of the European continent and is transmitted by direct contact with sick fish or contaminated water. Symptoms of the disease include red bleeding spots around the eyes and gills, swelling, and blood clots in body fat, viscera, and muscles (1, 2). So far, four main genotypes of this virus have been identified, and the differences in the nucleotide sequence and the genes coding for the nucleocapsid and glycoproteins distinguish the genotypes phylogenetically (3). The symptoms of this disease have three states: acute, chronic, and nervous. In the acute form, severe mortality occurs among lethargic and anorexic fish. Additionally, extensive bleeding is observed in the eyes, fins, and internal organs. In the chronic state, the mortality rate is lower, and the fish become dark in color and suffer from minor degrees of bleeding. Exophthalmia is a symptom of the chronic condition of this disease. In the nervous state, unusual swimming and convulsions are observed, but the mortality rate is very low (4).

VHSV, like other rhabdoviruses, has a single-stranded and negative-sense RNA genome. The genome of all rhabdoviruses contains 5 genes: nucleoprotein (N), polymerase-associated phosphoprotein (P), matrix protein (M), glycoprotein (G), and RNA-dependent RNA polymerase (L) (5). The VHS virus also has a non-virion (NV) gene located between the G and L genes in the genome. The NV gene is specific to *novirhabdoviruses* and encodes a non-structural protein that is not present in the VHS virion but is only seen in infected cells. The presence of the NV gene is necessary for pathogenicity in some strains, especially in rainbow trout. This gene is responsible for suppressing apoptosis and innate immune responses and is also required for efficient replication (6).

So far, various types of vaccines have been tested as a common strategy against viruses to deal with the hemorrhagic septicemia virus. For example, whole-virus inactivated vaccines, attenuated vaccines, DNA vaccines, and recombinant G protein-based vaccines have been developed *in vitro*. However, challenges such as production difficulty, safety, and high production costs are barriers to commercializing these vaccines. Additionally, several studies focused on compounds and drugs with antiviral activity against VHSV have been conducted, but none of these agents are completely effective. Therefore, the need for preventive strategies and strong, specific treatments against the infection of this virus is felt (7, 8).

RNA interference (RNAi) is a silencing mechanism that suppresses gene expression in various biological systems in the post-transcriptional stage with high specificity and efficiency. Suppression of the gene will cause the termination of its essential functions. For this reason, RNAi is considered an effective protection method against viral and bacterial pathogens. Based on previous studies, RNAi as a strategy with high potential can act against various diseases, including viral diseases (9). The RNA interference approach, which can target almost all viral genes, is considered a flexible and effective strategy to control viral infections. Specific suppression of the main cause of the disease in both prophylactic and curative phases is the special advantage of this treatment strategy. Also, some of the benefits of the RNAi strategy compared to conventional antiviral drugs are rapidity, ease of use, and specificity (10).

Two important RNAi molecules with functions in post-transcriptional gene regulation are genome-derived miRNAs and exogenous siRNAs. The mechanisms of action of siRNA and microRNA are largely similar through incorporation into the RNA-induced silencing complex (RISC). SiRISC usually exerts its function by targeting a specific mRNA, thereby degrading it. miRISC, on the other hand, targets several specific mRNAs, leading to mRNA degradation, translational inhibition, and rarely translational activation (11).

shRNA, as an RNA molecule with a short hairpin secondary structure, is one of the most important functional molecules for RNA interference. In recent years, this molecule has been considered an attractive therapeutic tool through the targeting of disease-specific genes. Delivery of this molecule through pcDNA or viral vectors to host cells, followed by its stable expression in the nucleus, leads to long-term gene silencing. shRNA has a potential advantage over siRNA by integrating into the host cell genome and creating a stable RNAi (12, 13).

In recent years, lentiviral vectors have been used as a very effective vector class for the delivery of shRNA molecules. These vectors are derived from viruses that lead to the stable expression of shRNA molecules in a wide range of cells through integration into the DNA of the host cell. Advantages such as high-efficiency infection of dividing and non-dividing cells, high titer vector stock, large insertion capacity, and low immunogenicity make lentiviral vectors an efficient delivery tool for therapeutic purposes (14, 15).

In the present study, considering the special role of the NV gene in VHS virus replication and pathogenicity, shRNAs designed against the NV gene were used to inhibit virus infection. After that, the production of lentivectors expressing shRNA molecules was done in the HEK293T cell line. Finally, the effect of these shRNA molecules on reducing the virus titer in the CS2-2 cell line was evaluated using the TCID50 test.

## 2 Materials and methods

### 2.1 Designing shRNA molecules against the VHSV-NV gene

At first, the NV gene sequence related to the VHSV Fil 3 strain was taken from the NCBI database. Then this sequence was aligned with the NV gene sequence belonging to other VHS virus strains. This step was done to obtain the conserved regions of the NV gene with the help of Clustal omega. Three online software including BLOCK-iT™ RNAi Designer, WI siRNA Selection Program, and siRNA Wizard^1^ were used to design the proposed shRNA sequences. shRNA molecules matching conserved regions were selected for further analysis by aligning all proposed molecules to these regions. Each of these software evaluates various aspects such as efficiency, potential off-target effects, and the risk of potential immune responses. One of the steps to select the most efficient shRNA sequences was to examine the secondary structure of the NV mRNA to ensure access to its target sites. For this purpose, we used http://www.unafold.org/ to obtain the secondary structure of the target mRNA and then selected only shRNAs that were complementary to the loop regions for further selection. Those that targeted the stem regions were eliminated due to steric hindrance. Since non-targeting of the host genome is a key and important consideration when designing shRNA sequences, the proposed shRNAs were also blasted with fish mRNAs. The shRNAs that had 15 nucleotides or more homology to the host genome were eliminated. Finally, a final ranking was performed based on several key criteria including optimized GC content (40–60%), location of target sites in mRNA (desirable regions with minimal spatial interference), and the number of off-target interactions predicted by the design tools (16). Finally, three shRNA molecules with the highest score as the best candidate for gene targeting were finalized and synthesized by Bioneer Co.

### 2.2 Production of lentiviral plasmids expressing anti-VHSV-NV shRNAs

The pCDH-CMV-MCS-EF1-cGFP-T2A-Puro lentiviral plasmid (provided by Bonbiotech) was used as a transfer plasmid in the third-generation lentiviral packaging system. This 8,220 bp plasmid contains a puromycin resistance cassette and a CopGFP gene to ensure the efficiency of the transfection process. During the cloning process, the lentiviral plasmid was double-digested by *EcoR*I and *BamH*I enzymes, and then the ligation of the proposed shRNA sequences was done separately using the *T4* DNA Ligase enzyme. Before the ligation step, shRNA fragments were prepared by annealing the sense and antisense strands of the synthesized single-stranded oligomers. In the next step, the *Escherichia coli DH5α* strain was applied to transform plasmids carrying shRNAs. The heat shock method was used for transforming ligation products in DH5α bacteria. Following the transformation, the precision of the cloning was confirmed by a colony PCR reaction and subsequent sequencing. Eventually, recombinant lentiviral plasmids were extracted from *DH5α* bacteria. It should be noted that in this study, another vector named “pEZX-MR03” was used, which possesses an EGFP gene controlled by the CMV promoter. Since the structure of this plasmid lacks shRNA sequences, it was used as a mock in the transfection process (17).

### 2.3 Cell line propagation

In this study, the HEK293T cell line (ATCC number: CRL-1573) was used for transfecting and packaging of third-generation lentiviral plasmids. Another cell line that was used is the CS2-2 cell line (ATCC Number: IBRC C10190-3), which was employed to investigate the lentivirus and VHSV challenge as well as the TCID50 test. The origin of this cell line is the epithelial cells from the fin tissue of salmon in the Caspian Sea.

### 2.4 Virus propagation

In order to investigate the inhibitory effect of designed shRNA molecules on the proliferation of hemorrhagic septicemia virus, the VHSV-Fil3 standard strain (French National Institute for Agricultural Research, France) was utilized. The titer of the viral stock was determined to be 5/555×10^+1.5^.

### 2.5 Production of the third-generation lentiviral packaging system

Initially, HEK293T cells were cultured in 6 cm plates and then transfected with one transfer vector and two other vectors using the calcium phosphate method. Following the guidelines from Bonbiotech company, a DNA mixture containing 8 μg of pCDH recombinant plasmid (or pEZX-MR03 as a mock), 5 μg of pMD2G -(a VSV-G envelope expressing vector), and 8 μg of psPAX2- (a packaging vector) was co-transfected into HEK293T cells with a confluency of approximately 80%. Fluorescent microscopy was then performed on the cells to evaluate the expression of the GFP gene in the transfer plasmid. For this purpose, the transfected cells were observed and photographed by a fluorescent microscope 48 and 72 h after transfection. If sufficient GFP was observed, the supernatants containing the lentivectors were collected from the plates. The supernatants were then centrifuged at 1000 g for 15 min to remove the cell debris. These lentivectors were subsequently stored at −70°C for the challenge with VHSV (18).

### 2.6 Infection of CS2-2 cells with lentiviruses and VHSV inoculation

At first, a monolayer culture of CS2-2 cells was prepared, then 200,000 trypsinized cells were seeded in 6 cm plates. After adding fresh and warm DMEM medium (Gibco, America, Catalog No. 116–12800) +1x penicillin–streptomycin (Sigma, America Catalog No. 116–12,800) + 10٪ FBS (Gibco, America, Catalog No. 106–10270) to the cells, the plates were incubated overnight at 37°C and 5% CO2. Since the transduction process requires approximately 70% confluence, the level of cell confluency in each plate was checked. The following day, the culture medium of the plates was removed, and 2.25 mL of medium containing lentiviruses expressing shRNAs and scrambled lentivirus were added to the plates with an MOI of 2. The cells were infected once again with lentiviruses 24 h after the initial infection. Observing and photographing the cells using a fluorescent microscope was performed 48 and 72 h after infection with lentiviruses. If sufficient GFP was observed, the challenge with VHSV was performed by inoculating 123 μL of VHSV into each 6 cm plate. Seventy two hours after infection with VHSV, the cells’ phenotype was examined in terms of the development of cytopathic effects (CPE). In this assay, cells infected only with VHSV were considered the positive control. Additionally, cells infected with the scrambled lentivector, as well as those without any viral infection, were used as negative controls (19). For statistical analysis, differences in mean TCID50 values between groups were assessed using an independent t-test in SPSS software (version 22), with statistical significance set at *p* < 0.05.

### 2.7 Calculation of VHSV titer after challenge

In order to prepare the viral dilutions required for the TCID50 test, the supernatants of the test and control plates obtained from the previous step were centrifuged (15 min, 1,000 g). 10,000 CS2-2 cells were seeded in the wells of the 96-well plate, then 100 μL of Lebovitz’s L15 medium (Gibco, America, Catalog No. 11415049) +10% FBS (Gibco, America, Catalog No. 106–10270) +1X penicillin–streptomycin (Sigma, America Catalog No. 116–12800) +1 mM sodium pyruvate (DENA zist Assia, Iran, Catalog No. S-5039) +1 mM L-glutamine (DENA zist Assia, Iran, Catalog No. S-8061) +10 μL/μg epidermal growth factor (Thermo Fisher Scientific, United States, Catalog No. PHG0314) and 1 μΜ non-essential amino acid (Gibco, America, Lot No. 2055325) were added to each well. A 1:10^−5^ dilution series of the virus was prepared using Lebowitz medium and inoculated into the wells. Morphological changes and cytopathic effects on cells were investigated for 1 week. The titer of the virus was then determined using the Reed-Muench method. In addition to four replicates for each viral dilution, positive control (cells infected only with VHSV), negative control (cells infected with scrambled lentivector), and cells without any inoculation were included (20). SPSS version 22 software and *t*-test and ANOVA were used to analyze the data and determine the statistical significance of the differences between the groups.

## 3 Results

### 3.1 Selecting and screening the shRNA molecules

All the shRNA molecules designed by the mentioned software were screened to select effective shRNAs. After removing a series of undesirable shRNAs during the investigations, the remaining shRNAs were scored based on sequence specificity, GC percentage, binding site to target mRNA secondary structure, number of off-targets, probability of inducing an immune response, and the software’s own rank. Allocating points to them based on their performance in each of these fields made it possible to select the three final shRNA molecules with the highest score and the best characteristics. The sequence of three final shRNA molecules selected for synthesis, named VHS-ShRNA 1, VHS-ShRNA 2, and VHS-ShRNA 3, are shown in Table 1. Preparation of lentivectors expressing shRNAs.

**Table 1:** The anti-NV shRNA oligonucleotides sequences.

Name	shRNA sequence
VHS-shRNA1	AATTCGCTCGACATGAGGATAAGTCTTCAAGAGAGACTTATCCTCAT
VHS-shRNA2	AATTCCACAGACTCAAATTTGACCCAAGTCAAGAGCTTGGGTCAAATT
VHS-shRNA3	AATTCCCTTGATCGGTCGGACATATCAAGAGTATGTCCGACCGATCAAGGG

**Figure 1:**
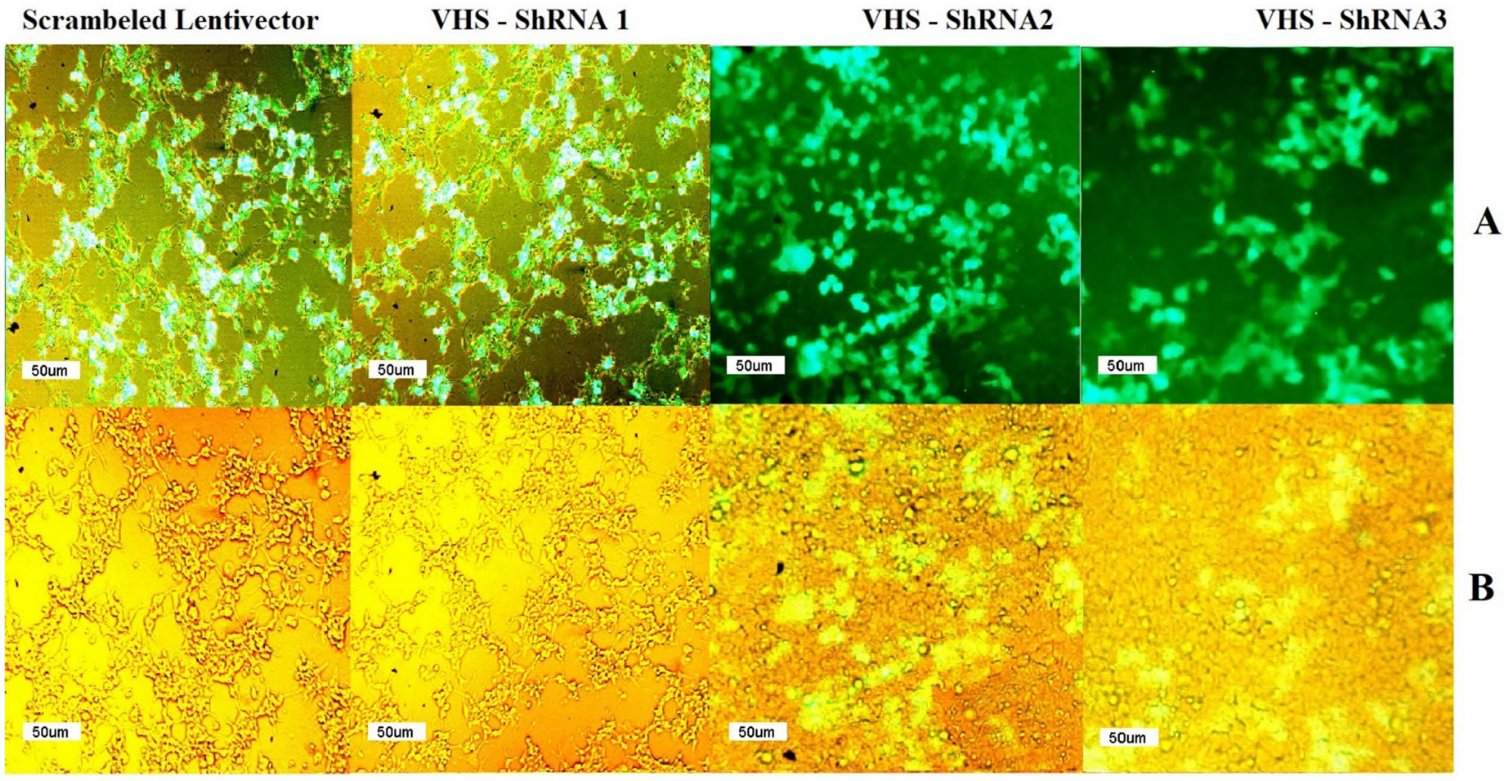
**(A)** GFP observed by fluorescent microscope after transfection of HEK293T cells with psPAX and PMD2.G as packaging vectors and pEZX-MR03 (as a mock), pCDH carrying VHS-ShRNA1, pCDH carrying VHS-ShRNA2, and pCDH carrying VHS-ShRNA3, respectively. **(B)** Identical images obtained using a light microscope.

Fluorescent microscopy confirmed the successful production of shRNA-expressing lentivectors by observing GFP in HEK 293 T cells transfected with the pCDH plasmids carrying VHS-ShRNA 1, VHS-ShRNA 2, VHS-ShRNA 3, and the mock vector. Since the GFP gene is located downstream of the cloned shRNAs in the pCDH plasmid, the expression of this gene indicates the expression of shRNAs in the cells (Figure 1).

### 3.2 The challenge of lentivectors expressing shRNAs with VHSV

**Figure 2:**
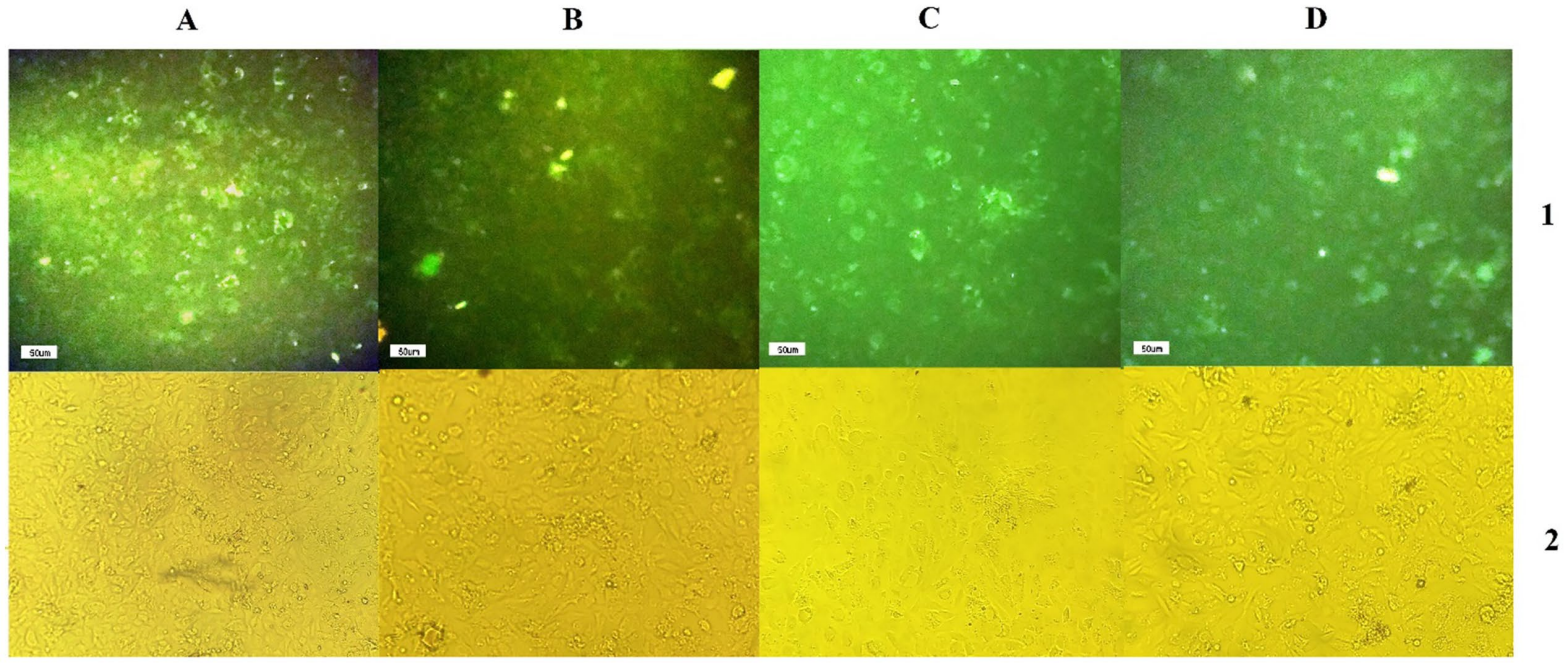
**(A1,B1,C1,D1)** GFP expression by infected CS2-2 cells with scrambled lentivector, lentivector expressing VHS-ShRNA1, VHS- ShRNA2, and VHS-SHRNA 3, respectively, **(A2,B2,C2,D2)** identical images obtained using a light microscope.

**Figure 3:**
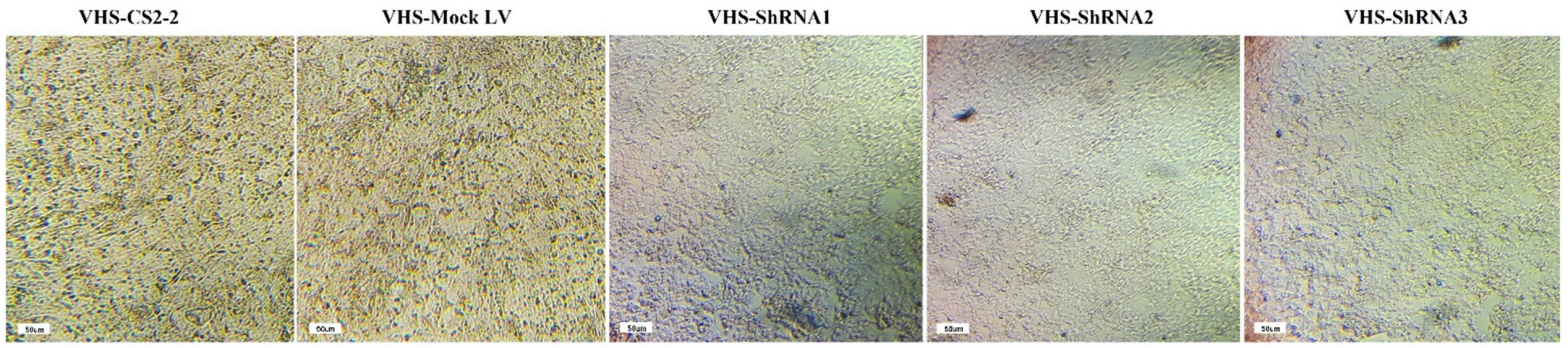
Development of cytopathic effects in CS2-2 cells after *VHSV* challenge and lentivectors: The presence of cytopathic effects in cells infected with *VHSV* and cells infected with scrambled lentivector and VHSV. Lack of cytopathic effects in cells infected with *VHSV* and lentivectors expressing VHS-ShRNA1, VHS-ShRNA2, and VHS-ShRNA3, respectively.

The efficiency of shRNA expression was demonstrated through fluorescent microscopy and evaluation of GFP expression in CS2_2 cells infected with lentivectors expressing shRNA and the scrambled lentivector (Figure 2). Examining CS2_2 cells in terms of phenotypic changes 72 h after the VHSV challenge showed that in cells infected with anti-VHSV shRNAs, the development of cytopathic effects was significantly reduced. Conversely, cells infected with only VHSV and cells infected with scrambled lentivector and VHSV showed a high degree of morphological changes due to viral infection (Figure 3).

### 3.3 TCID50 test

**Figure 4:**
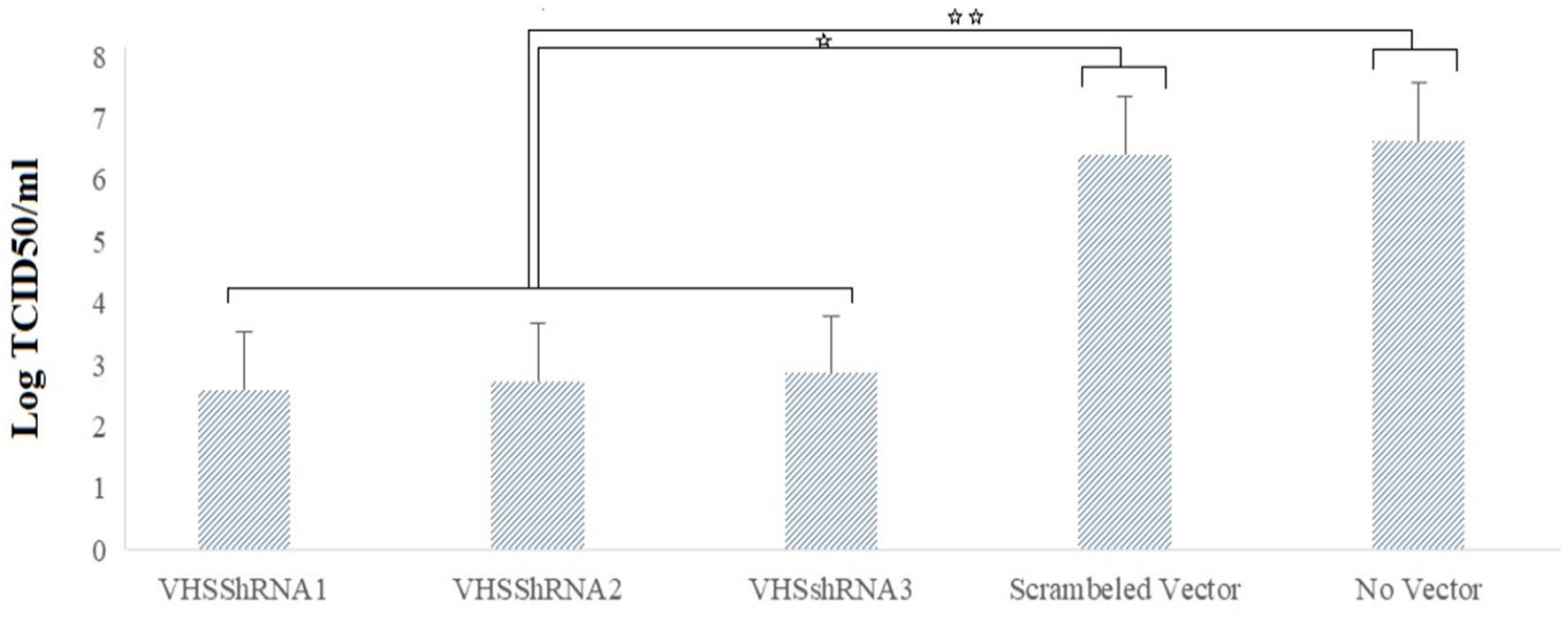
Reduction of Log TCID50 of VHS virus due to the inoculation of lentivectors expressing shRNA molecules. *p < 0.05, **p < 0.001.

A TCID50 test was performed to calculate the VHSV titer after the challenge, and the results indicated the beneficial effect of shRNAs in reducing the viral yield. VHS_shRNA1, VHS_shRNA2, and VHS_shRNA3 reduced the virus titer by 99.99, 99.99, and 99.98%, respectively, compared to cells infected with VHSV. This decrease in titer was approximately 99.98, 99.98, and 99.97%, respectively, compared to cells infected with scrambled lentivector and VHSV (Figure 4). Between the samples treated with ShRNAs and the control groups, the difference in Log TCID50/mL was statistically significant, but no statistically significant difference was found within the groups treated with ShRNAs.

## 4 Discussion

Viral Hemorrhagic Septicemia Virus (VHSV) is the cause of a highly fatal systemic disease that results in widespread losses among fish and other aquatic species. Introduction of pathogen-free water to farms, quarantine of potential carrier fishes, disinfection of used tools, continuous health checks of fish, following the biosecurity guidelines by farm workers, and administration of immune system stimulants are some of the strategies used to control the spread of VHSV in fish farms. Developing effective vaccines against VHSV, such as inactivated vaccines, is challenging due to its weak immunogenicity. It can be mentioned that despite continuous efforts in recent years, vaccines and antiviral compounds designed to combat VHSV have not been commercialized or used on a large scale (21). A commercial vaccine has been in use in Korea since 2019, but it is not effective for all olive flounders and only immunizes fish larger than 15 cm (22).

The science of genetics has the potential to suppress viral replication and, as a result, inhibit harmful viruses with a relatively ideal method through gene inhibition. In recent years, there has been a growing interest in using short hairpin RNAs (shRNAs) instead of siRNA and miRNA for therapeutic purposes and to create stable RNA interference (RNAi) (23). While RNA interference by siRNA has a temporary function, shRNA-based RNAi is a more suitable option for long-term gene silencing of pathogens. The integration of shRNAs into the genome of the host cell and their subsequent transfer to daughter cells maintains the stability of their function in several generations (24). Therefore, considering the significant advantages of shRNAs, we utilized these molecules to increase the efficiency of gene silencing and inhibit VHS virus replication.

The use of RNA interference strategy as an inhibitory tool to control aquatic viruses has been investigated in various studies. For example, Dang et al. proved that siRNAs designed against the major capsid protein (MCP) gene in Red sea bream iridovirus (RSIV) can successfully reduce viral particle production. In cells treated with siRNAs, the level of MCP gene expression decreased by 55.2 and 97.1% at 48 and 72 h after infection, respectively. This study suggested that nucleic acid-based drugs can effectively manage viral infections in the aquaculture industry (25). Targeting major capsid protein (MCP) to inhibit Rock bream iridovirus (RBIV) showed promising results in a study by Zenke et al. In this study, the synthesis and transfection of siRNA expression vectors into grunt fin cells (GF cells) led to a decrease in the mRNA levels of MCP, subsequently increasing the resistance of cells to the virus (26). In another study, in order to inhibit *in vitro* a lethal virus in carp farming called Cyprinid herpesvirus 3, Gotesman et al. used designed siRNAs against thymidine kinase and DNA polymerase genes. The measurement of viral replication by qPCR showed the greater effect of siRNAs targeting the DNA polymerase gene in reducing the viral titer (27). The study conducted by Fouad et al. on the use of siRNAs targeting the RdRp gene of the spring viremia of carp virus (SVCV) yielded successful results. Transfection of three different siRNAs for gene targeting in EPC cells led to a reduction in SVCV replication (28).

In addition to the fish farming industry, viral infections have posed threats to the shrimp farming industry, and because shrimp do not have an adaptive immune system, the use of protein vaccines does not have enough effect against shrimp virus infections. Therefore, shrimp health managers have been exploring the potential of RNAi molecules such as dsRNA and siRNA to inhibit the proliferation of these viruses (29). By inhibiting the White spot syndrome virus (WSSV) as one of the most harmful shrimp pathogens *in vivo*, Xu et al. demonstrated that RNAi can be developed as a therapeutic strategy against this pathogen through gene targeting. They treated *Penaeus japonicus* shrimp with vp27_siRNA to knock out the main envelope protein gene, and the final result indicated a reduction in mortality in the treated shrimp (30). Inhibition of another fetal shrimp virus called Yellow head virus (YHV) was confirmed in a study by Saksmerprome et al. The Development of a hairpin expression vector to produce long dsRNAs inhibited RdRP and successfully induced protection in infected shrimp (31). The silencing of the vp9 gene of the WSSV in the study conducted by Alenton et al. proved that sequence-specific dsRNAs can be considered an effective tool to inhibit this virus. In this *in vivo* study, the survival of three infected shrimp species increased after treatment with vp9_dsRNA (32).

In the case of VHSV, the application of RNA interference-based strategies to inhibit viral infection by knocking down key genes has attracted attention. It should be noted that there are several researches focused on designing and applying interfering molecules such as siRNA, dsRNA, and miRs *in vitro*, and sometimes in vivo, to suppress VHSV. For example, Bohle et al. used DsiRNAs to target the VHSV nucleocapsid N in salmonid fish CHSE_214 cells. The DsiRNAs designed in this study successfully reduced the mRNA level and replication of VHSV (33). In another study, Kim et al. evaluated the inhibition of VHSV replication by a vector producing long double-stranded RNA. This study demonstrated the inhibitory effect of dsRNA-based RNAi by targeting the VHSV G gene in EPC and CHSE 214 cells (34). In another study, inhibition of VHSV proliferation in EPC cells using three different siRNAs was performed by Schyth et al. The results indicated specific interference by siRNAs that targeted the viral glycoprotein gene (35). In another study by Au et al., they investigated the knockdown of VHSV-IVa and several other viral models by long dsRNAs and an RNAi-like mechanism. They explored the potential of dsRNAs to induce antiviral responses through a mechanism unrelated to the interferon-dependent response and successfully demonstrated this (36). The antiviral activity of miR-155 against VHSV infection was investigated *in vivo* by Liyanage et al. They encapsulated the dre-miR-155 mimic in chitosan nanoparticles, and the final results showed a decrease in virus copy number. The high expression of miR-155, along with the regulation of immune system responses, led to the protection of zebrafish against this virus infection (37).

In this study, we designed specific shRNA molecules as a gene knockdown tool in VHSV and used a third-generation lentiviral vector to effectively deliver these molecules to fish cell lines. Unfortunately, we did not find any research on the use of lentivector-based shRNAs to inhibit VHSV, but there have been studies focused on the induction of RNAi with the help of shRNA expression constructs. For example, Kim et al. inhibited this virus in EPC cells using a shRNA-producing construct. In their study, shRNA expressed by the fugo_U6 promoter knocked down the glycoprotein gene in VHSV (38). Vector-based shRNA succeeded in inhibiting VHSV replication *in vitro* as reported by Clarke et al. The simultaneous targeting of G and L genes by the designed shRNAs showed more effect of shRNAs targeting viral glycoprotein than shRNAs targeting viral polymerase. In this study, some of the shRNAs used led to a decrease in VHSV titer by 2–4 logs (39).

Because choosing the right vector is a very important issue that impacts the entire research process, it is necessary to consider various factors when selecting an efficient vector. The vector used in this research is a third-generation lentiviral vector with a capacity of approximately 8 kb. These vectors infect a wide range of hosts and are considered safer for therapeutic use due to containing only 10% of the viral genome sequence (40). Another crucial aspect of vectors is their ability to not induce disease or immune responses in the host, which lentivectors possess this characteristic. Another advantage of these vectors is their ability to remove the U3 region from 3’LTR, which leads to the creation of the SIN lentivector. As soon as lentiviral plasmids enter the cell, they lose their transcription ability. Thus, the probability of promoter interference and activation of genes adjacent to the integration site is very low (41).

The reason for choosing the NV gene for targeting is its main role in viral pathogenicity and replication. When designing shRNA for gene targeting, the first thing to consider is the selection of appropriate target transcripts. It is important to note that the target transcript is conserved among the different strains reported for a particular virus (16). Therefore, in this study, we obtained the highly conserved regions of the NV gene in VHSV before designing the shRNA sequences.

The current study’s use of RNA interference was restricted to the cell culture system. However, we anticipate that RNA interference (RNAi) treatments will demonstrate promising results for *in vivo* investigations based on previous studies. For example, several studies have been published related to the creation of transgenic animals with the ability to express short hairpin RNA (shRNA) against various animal viruses. The successful generation of transgenic pigs using shRNAs targeting the viral polymerase 3D protein and nonstructural protein 2B in Foot-and-Mouth Disease Virus (FMDV) (42), the production of FMDV-resistant transgenic goats in a similar manner through inhibition of the 3D pol gene (43), the development of a genetically engineered mouse resistant to FMDV through shRNA expression, the creation of a goat expressing shRNA directly against the prion protein responsible for transmissible spongiform encephalopathies, and the development of a calf capable of expressing an anti-prion shRNA are some of the illustrations of the potential uses for interfering RNAs *in vivo* (44).

The current study’s findings validated its further implementation in in vivo and field situations. It is crucial to note that even with the proof of RNAi’s efficiency and positive results of related studies, the application of this method in vivo will require more research and investigation to fully determine its side effects on the treated organism. Despite facing numerous challenges, the deeper understanding of RNAi mechanisms, thorough examination of regulatory processes in animals and disease progression, and advancements in synthetic chemistry and genome engineering have presented promising prospects for utilizing RNAi to improve animal welfare.

## 5 Conclusion

The study targets the non-structural NV gene of VHSV, essential for viral replication and pathogenesis, which has not been extensively addressed in previous RNAi strategies. By focusing on this gene, it presents a novel therapeutic target. The research employs third-generation lentiviral vectors to introduce new short hairpin RNAs (shRNAs), allowing for permanent integration into the host genome and extended gene silencing, significantly improving upon traditional transfection methods. The shRNA constructs were rigorously validated through bioinformatics to ensure specificity and minimize off-target effects, setting this work apart from earlier studies. The current study’s findings indicate that anti-VHSV-NV shRNAs exhibited strong antiviral properties and prevented VHSV multiplication in CS2_2 cells; hence, lentivirus-mediated RNA interference (RNAi) can be utilized as an advantageous approach to avoid VHSV infection. Ultimately, the findings enhance the understanding of RNAi techniques for treating VHSV and pave the way for future research on RNAi applications against other viral diseases in aquatic animals, potentially leading to transgenic organisms or innovative vaccine strategies.

## Data Availability

The data presented in the study are deposited in the Figshare repository: https://doi.org/10.6084/m9.figshare.28665077.v1.

## Footnotes

^1^ www.invivogen.com/sirna-wizard

## References

[1] Baillon L Mérour E Cabon J Louboutin L Vigouroux E Alencar ALF. The viral hemorrhagic septicemia virus (VHSV) markers of virulence in rainbow trout (*Oncorhynchus mykiss*). Front Microbiol. (2020) 11:574231. doi: 10.3389/fmicb.2020.574231, PMID: 33193184 PMC7606196

[2] Ahmadivand S Soltani M Mardani K Shokrpoor S Rahmati-Holasoo H Mokhtari A. Isolation and identification of viral hemorrhagic septicemia virus (VHSV) from farmed rainbow trout (*Oncorhynchus mykiss*) in Iran. Acta Trop. (2016) 156:30–6. doi: 10.1016/j.actatropica.2016.01.005, PMID: 26777311

[3] Hwang JY Lee UH Heo MJ Kim MS Jeong JM Kim SY. Naturally occurring substitution in one amino acid in Vhsv phosphoprotein enhances viral virulence in flounder. PLoS Pathog. (2021) 17:e1009213. doi: 10.1371/journal.ppat.1009213, PMID: 33465148 PMC7845975

[4] Woo P Bruno D. Fish diseases and disorders. Viral, Bacterial and Fungal Infections: Cabi (2011) 3.

[5] Einer-Jensen K Ahrens P Forsberg R Lorenzen N. Evolution of the fish Rhabdovirus viral Haemorrhagic Septicaemia virus. J Gen Virol. (2004) 85:1167–79. doi: 10.1099/vir.0.79820-0, PMID: 15105533

[6] Ammayappan A Vakharia VN. Nonvirion protein of *Novirhabdovirus* suppresses apoptosis at the early stage of virus infection. J Virol. (2011) 85:8393–402. doi: 10.1128/jvi.00597-11, PMID: 21653667 PMC3147959

[7] Kim MS Kim KH. Genetically engineered viral hemorrhagic septicemia virus (VHSV) vaccines. Fish Shellfish Immunol. (2019) 95:11–5. doi: 10.1016/j.fsi.2019.10.031, PMID: 31622675

[8] Zhang W Chen X Yu F Li F Li W Yi M. Α-lipoic acid exerts its antiviral effect against viral hemorrhagic septicemia virus (VHSV) by promoting upregulation of antiviral genes and suppressing VHSV-induced oxidative stress. Virol Sin. (2021) 36:1520–31. doi: 10.1007/s12250-021-00440-5, PMID: 34510367 PMC8435143

[9] Tan FL Yin JQ. RNAi, a new therapeutic strategy against viral infection. Cell Res. (2004) 14:460–6. doi: 10.1038/sj.cr.7290248, PMID: 15625012 PMC7092015

[10] Kang H Ga YJ Kim SH Cho YH Kim JW Kim C. Small interfering RNA (siRNA)-based therapeutic applications against viruses: principles, potential, and challenges. J Biomed Sci. (2023) 30:88. doi: 10.1186/s12929-023-00981-9 37845731 PMC10577957

[11] Traber GM Yu AM. RNAi-based therapeutics and novel RNA bioengineering technologies. J Pharmacol Exp Ther. (2023) 384:133–54. doi: 10.1124/jpet.122.001234, PMID: 35680378 PMC9827509

[12] Goel K Ploski JE. RISC-y business: limitations of short hairpin RNA-mediated gene silencing in the brain and a discussion of CRISPR/CAS-based alternatives. Front Mol Neurosci. (2022) 15:914430. doi: 10.3389/fnmol.2022.914430, PMID: 35959108 PMC9362770

[13] Lundstrom K. Viral vectors applied for RNAi-based antiviral therapy. Viruses. (2020) 12:924. doi: 10.3390/v12090924, PMID: 32842491 PMC7552024

[14] Frank SB Schulz VV Miranti CK. A streamlined method for the design and cloning of shRNAS into an optimized dox-inducible lentiviral vector. BMC Biotechnol. (2017) 17:24. doi: 10.1186/s12896-017-0341-x, PMID: 28245848 PMC5331646

[15] Deng L Liang P Cui H. Pseudotyped lentiviral vectors: ready for translation into targeted cancer gene therapy? Genes Dis. (2023) 10:1937–55. doi: 10.1016/j.gendis.2022.03.007, PMID: 37492721 PMC10363566

[16] Asadi Samani L Saffar B Mokhtari A Arefian E. Lentivirus expressing shRNAs inhibit the replication of contagious ecthyma virus by targeting DNA polymerase gene. BMC Biotechnol. (2020) 20:18. doi: 10.1186/s12896-020-00611-4, PMID: 32293394 PMC7092477

[17] Cockrell AS Kafri T. Gene delivery by lentivirus vectors. Mol Biotechnol. (2007) 36:184–204. doi: 10.1007/s12033-007-0010-8, PMID: 17873406

[18] Barde I Salmon P Trono D. Production and titration of lentiviral vectors. Curr Protoc Neurosci. (2010) 53:Unit 12.10. doi: 10.1002/0471142301.ns0421s53, PMID: 20938923

[19] Sastry L Johnson T Hobson MJ Smucker B Cornetta K. Titering lentiviral vectors: comparison of DNA, RNA and marker expression methods. Gene Ther. (2002) 9:1155–62. doi: 10.1038/sj.gt.3301731, PMID: 12170379

[20] Ramakrishnan MA. Determination of 50% endpoint titer using a simple formula. World J Virol. (2016) 5:85–6. doi: 10.5501/wjv.v5.i2.85, PMID: 27175354 PMC4861875

[21] Mohammadisefat P Zorriehzahra M Adel M Chamjangali Z Jabbari M Eftekhari A. Viral hemorrhagic septicemia virus (VHSV), past, present and future: a review. Int Aquat Res. (2023):15. doi: 10.22034/IAR.2023.1983457.1424

[22] Mendis WRH Lim J-W Kim G-W Kang SY. Antiviral activity of the Coumarin derivative Scoparone against viral hemorrhagic septicemia virus in vitro and in the olive flounder *Paralichthys olivaceus*. Aquaculture. (2024) 585:740703. doi: 10.1016/j.aquaculture.2024.740703

[23] Burnett JC Rossi JJ Tiemann K. Current progress of siRNA/shRNA therapeutics in clinical trials. Biotechnol J. (2011) 6:1130–46. doi: 10.1002/biot.201100054, PMID: 21744502 PMC3388104

[24] Subramanya S Kim SS Manjunath N Shankar P. RNA interference-based therapeutics for human immunodeficiency virus HIV-1 treatment: synthetic siRNA or vector-based shRNA? Expert Opin Biol Ther. (2010) 10:201–13. doi: 10.1517/14712590903448158, PMID: 20088715 PMC3745298

[25] Dang LT Kondo H Hirono I Aoki T. Inhibition of red seabream iridovirus (RSIV) replication by small interfering RNA (siRNA) in a cell culture system. Antivir Res. (2008) 77:142–9. doi: 10.1016/j.antiviral.2007.10.007, PMID: 18037509

[26] Zenke K Nam YK Kim KH. Development of Sirna expression vector utilizing rock bream Β-actin promoter: a potential therapeutic tool against viral infection in fish. Appl Microbiol Biotechnol. (2010) 85:679–90. doi: 10.1007/s00253-009-2177-3, PMID: 19685238

[27] Gotesman M Soliman H Besch R El-Matbouli M. In vitro inhibition of cyprinid Herpesvirus-3 replication by RNAi. J Virol Methods. (2014) 206:63–6. doi: 10.1016/j.jviromet.2014.05.022, PMID: 24893110 PMC4106878

[28] Fouad AM Soliman H Abdallah ESH Ibrahim S El-Matbouli M Elkamel AA. In-vitro inhibition of spring viremia of carp virus replication by RNA interference targeting the RNA-dependent RNA polymerase gene. J Virol Methods. (2019) 263:14–9. doi: 10.1016/j.jviromet.2018.10.008, PMID: 30336160

[29] Itsathitphaisarn O Thitamadee S Weerachatyanukul W Sritunyalucksana K. Potential of RNAi applications to control viral diseases of farmed shrimp. J Invertebr Pathol. (2017) 147:76–85. doi: 10.1016/j.jip.2016.11.006, PMID: 27867019

[30] Xu J Han F Zhang X. Silencing shrimp white spot syndrome virus (WSSV) genes by siRNA. Antivir Res. (2007) 73:126–31. doi: 10.1016/j.antiviral.2006.08.007, PMID: 17011052

[31] Saksmerprome V Charoonnart P Gangnonngiw W Withyachumnarnkul B. A novel and inexpensive application of RNAi technology to protect shrimp from viral disease. J Virol Methods. (2009) 162:213–7. doi: 10.1016/j.jviromet.2009.08.010, PMID: 19712700

[32] Alenton RR Kondo H Hirono I Maningas MB. Gene silencing of VP9 Gene impairs WSSV infectivity on *Macrobrachium rosenbergii*. Virus Res. (2016) 214:65–70. doi: 10.1016/j.virusres.2016.01.013 26811904

[33] Bohle H Lorenzen N Schyth BD. Species specific inhibition of viral replication using dicer substrate siRNAs (dsiRNAs) targeting the viral nucleoprotein of the fish pathogenic Rhabdovirus viral hemorrhagic septicemia virus (VHSV). Antivir Res. (2011) 90:187–94. doi: 10.1016/j.antiviral.2011.03.174, PMID: 21439327

[34] Kim MS Jee BY Cho MY Kim JW Jeong HD Kim KH. Fugu double U6 promoter-driven long double-stranded RNA inhibits proliferation of viral hemorrhagic septicemia virus (VHSV) in fish cell lines. Arch Virol. (2012) 157:1029–38. doi: 10.1007/s00705-012-1275-1, PMID: 22398916

[35] Schyth BD Lorenzen N Pedersen FS. Antiviral activity of small interfering RNAs: specificity testing using heterologous virus reveals interferon-related effects overlooked by conventional mismatch controls. Virology. (2006) 349:134–41. doi: 10.1016/j.virol.2006.01.009, PMID: 16480753

[36] Au SKW Portelli IV DeWitte-Orr SJ. Using long, sequence-specific dsRNA to knockdown inducible protein expression and virus production via an RNAi-like mechanism. Fish Shellfish Immunol. (2022) 131:945–57. doi: 10.1016/j.fsi.2022.10.061, PMID: 36351544

[37] Liyanage TD Nikapitiya C De Zoysa M. Chitosan nanoparticles-based in vivo delivery of MIR-155 modulates the viral haemorrhagic septicaemia virus-induced antiviral immune responses in zebrafish (*Danio RERIO*). Fish Shellfish Immunol. (2024) 144:109234. doi: 10.1016/j.fsi.2023.109234, PMID: 37984615

[38] Kim MS Kim KH. Inhibition of viral hemorrhagic septicemia virus replication using a short hairpin RNA targeting the G gene. Arch Virol. (2011) 156:457–64. doi: 10.1007/s00705-010-0882-y, PMID: 21184243

[39] Clarke BD McColl KA Ward AC Doran TJ. Shrnas targeting either the glycoprotein or polymerase genes inhibit viral haemorrhagic septicaemia virus replication in zebrafish Zf4 cells. Antivir Res. (2017) 141:124–32. doi: 10.1016/j.antiviral.2017.02.012, PMID: 28237822

[40] Segura MM Mangion M Gaillet B Garnier A. New developments in lentiviral vector design, production and purification. Expert Opin Biol Ther. (2013) 13:987–1011. doi: 10.1517/14712598.2013.779249, PMID: 23590247

[41] Schambach A Zychlinski D Ehrnstroem B Baum C. Biosafety features of lentiviral vectors. Hum Gene Ther. (2013) 24:132–42. doi: 10.1089/hum.2012.229, PMID: 23311447 PMC3581032

[42] Hu W Zheng H Li Q Wang Y Liu X Hu X. Shrna transgenic swine display resistance to infection with the foot-and-mouth disease virus. Sci Rep. (2021) 11:16377. doi: 10.1038/s41598-021-95853-3, PMID: 34385528 PMC8361160

[43] Li W Wang K Kang S Deng S Han H Lian L. Tongue epithelium cells from shRNA mediated transgenic goat show high resistance to foot and mouth disease virus. Sci Rep. (2015) 5:17897. doi: 10.1038/srep17897, PMID: 26671568 PMC4680861

[44] Bradford B Cooper C Tizard M Doran T Hinton T. RNA interference-based technology: what role in animal agriculture? Anim Prod Sci. (2016) 57:1–15. doi: 10.1071/AN15437, PMID: 39611015

