# Supplementary material for: Utilizing shRNA-expressing lentivectors for viral hemorrhagic septicemia virus suppression via NV gene targeting
Source: Front Vet Sci. 2025 Apr 4;12:1508470. doi: 10.3389/fvets.2025.1508470 (PMC12006114; doi:10.3389/fvets.2025.1508470)
Supplement: Supplementary file 1 [file Data_Sheet_1.zip › data/designing of the shRNA sequences.docx]

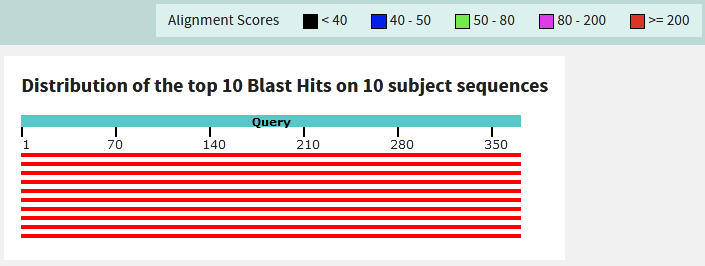


The results of the alignment of the NV gene sequences related to the strains with the most homology between the rhabdovirus family and the standard strain


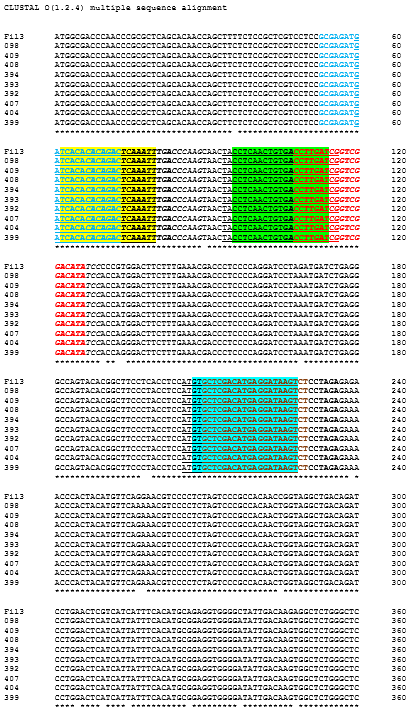


Alignment results obtained from the Clustal Omega online web server regarding the NV gene sequences of 10 selected strains. Conserved regions in the selected strains are shown as asterisks, and regions with potential for shRNA targeting are highlighted in color.


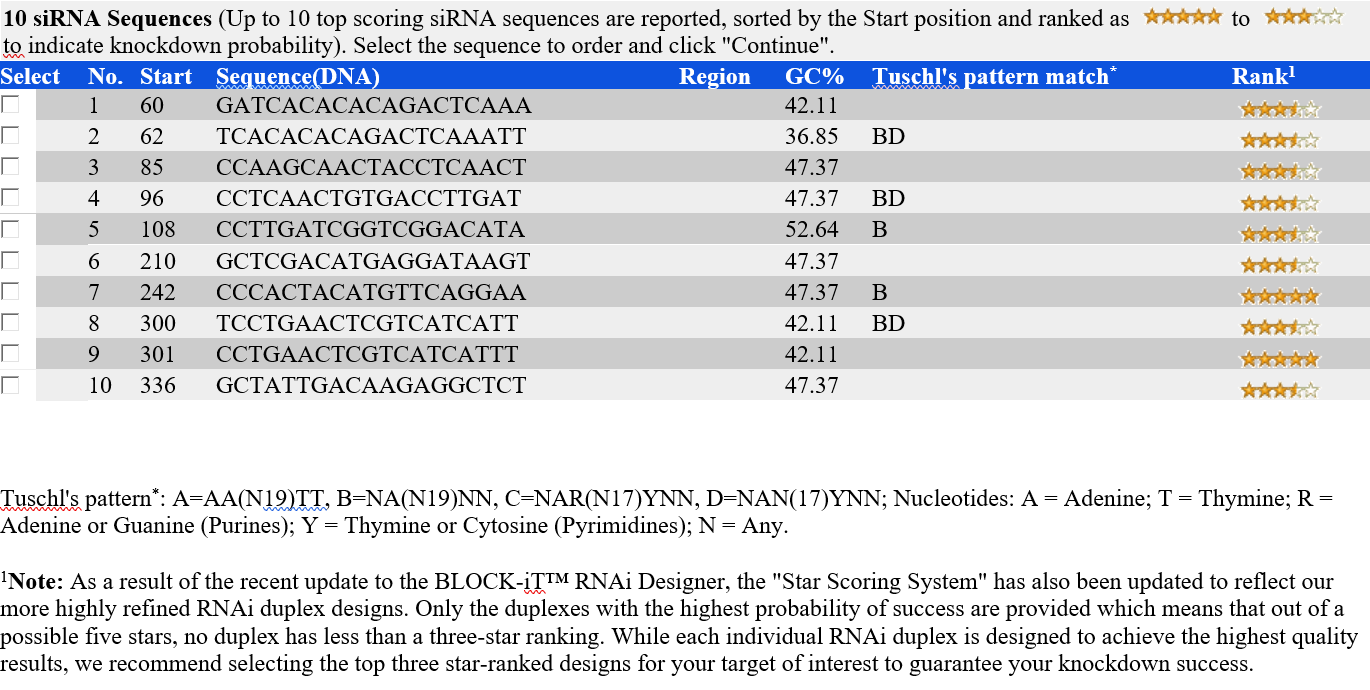


shRNAs designed by BLOCK-iT™ RNAi Designer online tool


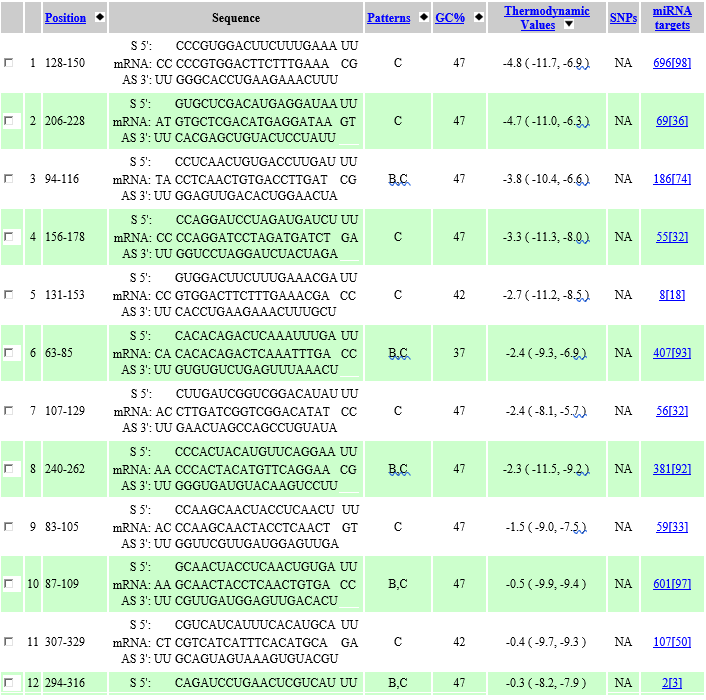


shRNAs designed by WI siRNA Selection Program online tool


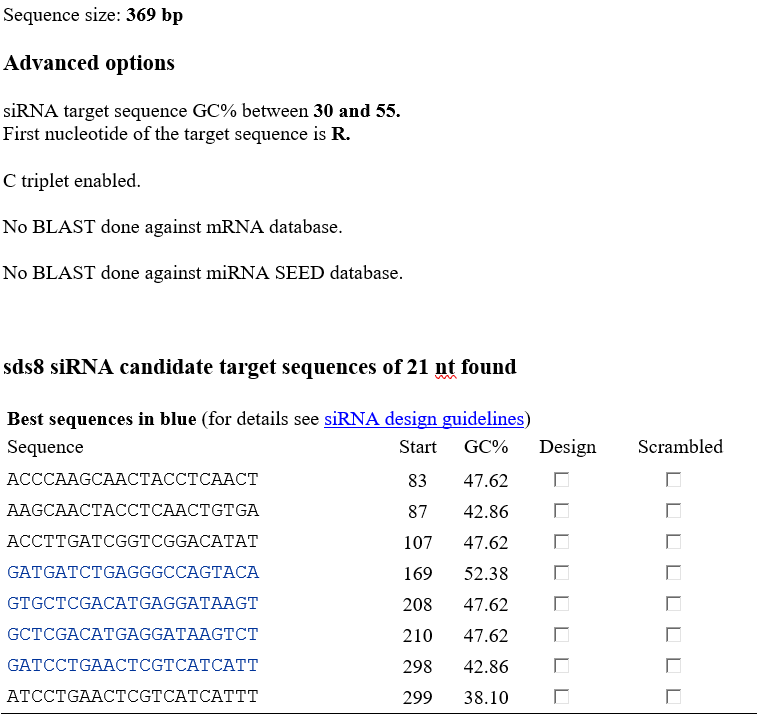


shRNAs designed by the online tool siRNA Wizard Software - Choose and design short hairpin RNAs (invivogen.com)


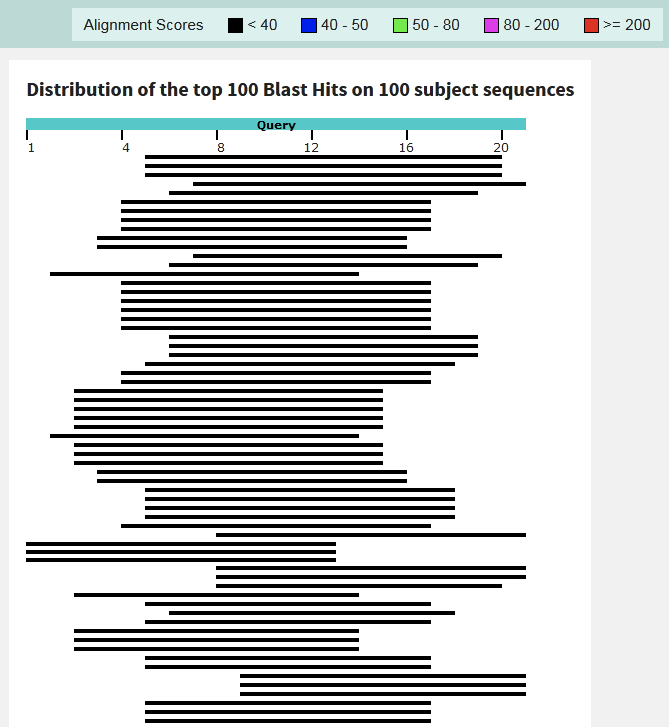


The results obtained from the alignment of VHSshRNA-1 with the fish genome by NCBI database

(The black color corresponding to the alignment indicates the absence of similarity between the VHSshRNA-1 sequence and the fish genome)


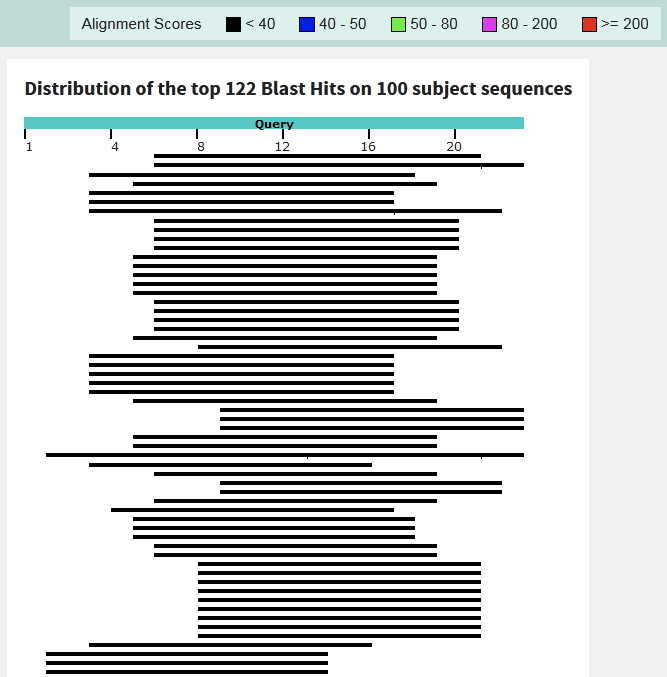


The results obtained from the alignment of VHSshRNA-2 with the fish genome by NCBI database

(The black color corresponding to the alignment indicates the absence of similarity between the VHSshRNA-2 sequence and the fish genome)


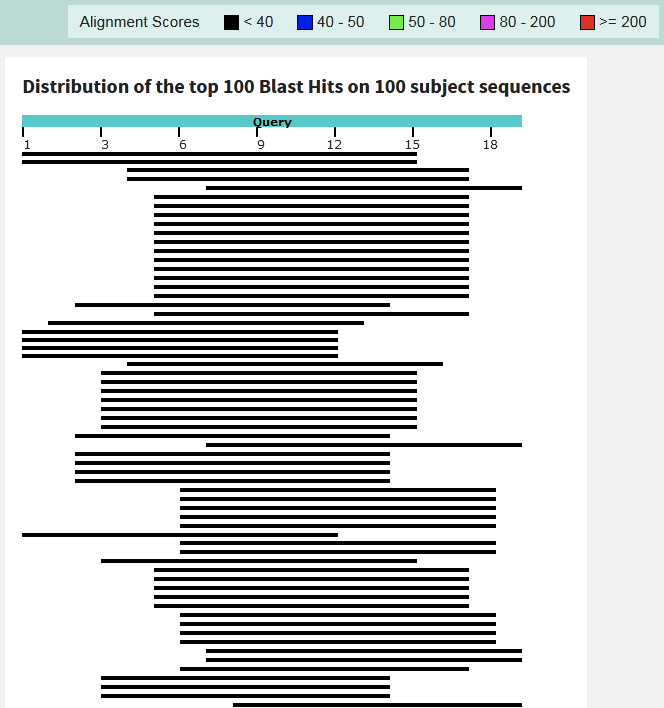


The results obtained from the alignment of VHSshRNA-3 with the fish genome by NCBI database

(The black color corresponding to the alignment indicates the absence of similarity between the VHSshRNA-3 sequence and the fish genome)
