# Supplementary material for: Utilizing shRNA-expressing lentivectors for viral hemorrhagic septicemia virus suppression via NV gene targeting
Source: Front Vet Sci. 2025 Apr 4;12:1508470. doi: 10.3389/fvets.2025.1508470 (PMC12006114; doi:10.3389/fvets.2025.1508470)
Supplement: Supplementary file 1 [file Data_Sheet_1.zip › data/TCID50 calculations.docx]

**Table 1. Reed-Muench table for determination of VHSV titer after inoculation of VHSshRNA-1 lentivector**

| **Percentage of infected wells** | $\frac{\mathbf{A}}{\mathbf{A+B}}$ | **Cumulative frequency of negative wells (B)** | **Cumulative frequency of infected wells (A)** | **number of infected wells** | **log dilution** |
| --- | --- | --- | --- | --- | --- |
| 80/0% | $\frac{\text{4}}{\text{5}}$ | 1 | 4 | $\frac{\text{3}}{\text{4}}$ | -1 |
| 20/0% | $\frac{\text{1}}{\text{5}}$ | 4 | 1 | $\frac{1}{4}$ | -2 |
| 0% | $\frac{\text{0}}{\text{8}}$ | 8 | 0 | $\frac{0}{4}$ | -3 |
| 0% | $\frac{\text{0}}{\text{12}}$ | 12 | 0 | $\frac{0}{4}$ | -4 |
| 0% | $\frac{\text{0}}{\text{16}}$ | 16 | 0 | $\frac{0}{4}$ | -5 |

**PD** = (Infection rate above 50% – 50%) / (Infection rate above 50%) – (Infection rate below 50%)

= $\frac{\text{80/0 – 50}}{\text{80/0 – 20/0}}\text{ }$= 0/50

**TCID50** = – [dilution with infection rate above 50% + The logarithmic unit of the dilution factor × (PD)]

= – [–1 + (–1) × (0/50)] = – [–1/50] = +1/50

$\frac{\text{10}^{\text{+1/50}}\text{ TCID50}}{\text{0/08}\text{ml}}\text{ }$= 1/25× 10^2/50^ TCID50

**Table 2. Reed-Muench table for determination of VHSV titer after inoculation of VHSshRNA-2 lentivector**

| **Percentage of infected wells** | $\frac{\mathbf{A}}{\mathbf{A+B}}$ | **Cumulative frequency of negative wells (B)** | **Cumulative frequency of infected wells (A)** | **number of infected wells** | **log dilution** |
| --- | --- | --- | --- | --- | --- |
| 100/0% | $\frac{\text{5}}{\text{5}}$ | 0 | 5 | $\frac{4}{4}$ | -1 |
| 25/0% | $\frac{\text{1}}{\text{4}}$ | 3 | 1 | $\frac{\text{1}}{\text{4}}$ | -2 |
| 0% | $\frac{\text{0}}{\text{7}}$ | 7 | 0 | $\frac{\text{0}}{\text{4}}$ | -3 |
| 0% | $\frac{\text{0}}{\text{11}}$ | 11 | 0 | $\frac{\text{0}}{\text{4}}$ | -4 |
| 0% | $\frac{\text{0}}{\text{15}}$ | 15 | 0 | $\frac{\text{0}}{\text{4}}$ | -5 |

**PD** = (Infection rate above 50% – 50%) / (Infection rate above 50%) – (Infection rate below 50%)

= $\frac{\text{100}\text{/0 – 50}}{\text{100/0 – 25/0}}\text{ }$= 0/66

**TCID50** = – [dilution with infection rate above 50% + The logarithmic unit of the dilution factor × (PD)]

= – [–1 + (–1) × (0/66)] = – [–1/66] = +1/66

$\frac{\text{10}^{\text{+1/}\text{66}}\text{ TCID50}}{\text{0/08}\text{ml}}\text{ }$= 1/25× 10^2/66^ TCID50

**Table 3. Reed-Muench table for determination of VHSV titer after inoculation of VHSshRNA-3 lentivector**

| **Percentage of infected wells** | $\frac{\mathbf{A}}{\mathbf{A+B}}$ | **Cumulative frequency of negative wells (B)** | **Cumulative frequency of infected wells (A)** | **number of infected wells** | **log dilution** |
| --- | --- | --- | --- | --- | --- |
| 83.33/0% | $\frac{5}{6}$ | 1 | 5 | $\frac{\text{3}}{\text{4}}$ | -1 |
| 40/0% | $\frac{\text{2}}{\text{5}}$ | 3 | 2 | $\frac{\text{2}}{\text{4}}$ | -2 |
| 0% | $\frac{\text{0}}{\text{7}}$ | 7 | 0 | $\frac{\text{0}}{\text{4}}$ | -3 |
| 0% | $\frac{\text{0}}{\text{11}}$ | 11 | 0 | $\frac{\text{0}}{\text{4}}$ | -4 |
| 0% | $\frac{\text{0}}{\text{15}}$ | 15 | 0 | $\frac{\text{0}}{\text{4}}$ | -5 |

**PD** = (Infection rate above 50% – 50%) / (Infection rate above 50%) – (Infection rate below 50%)

= $\frac{\text{8}\text{3.33}\text{ – 50}}{\text{83.33 – 40/0}}\text{ }$= 0/77

**TCID50** = – [dilution with infection rate above 50% + The logarithmic unit of the dilution factor × (PD)]

= – [–1 + (–1) × (0/77)] = – [–1/77] = +1/77

$\frac{\text{10}^{\text{+1/}\text{77}}\text{ TCID50}}{\text{0/08}\text{ml}}\text{ }$= 1/25× 10^2/77^ TCID50

**Table 4. Reed-Muench table for determination of VHSV titer after inoculation of Scrambled lentivector**

| **Percentage of infected wells** | $\frac{\mathbf{A}}{\mathbf{A+B}}$ | **Cumulative frequency of negative wells (B)** | **Cumulative frequency of infected wells (A)** | **number of infected wells** | **log dilution** |
| --- | --- | --- | --- | --- | --- |
| 100/0% | $\frac{\text{15}}{\text{15}}$ | 0 | 15 | $\frac{\text{4}}{\text{4}}$ | -1 |
| 100/0% | $\frac{\text{11}}{\text{11}}$ | 0 | 11 | $\frac{\text{4}}{\text{4}}$ | -2 |
| 87.5/0% | $\frac{\text{7}}{\text{8}}$ | 1 | 7 | $\frac{\text{3}}{\text{4}}$ | -3 |
| 66.66/0% | $\frac{\text{4}}{\text{6}}$ | 2 | 4 | $\frac{\text{3}}{\text{4}}$ | -4 |
| 16.66/0% | $\frac{\text{1}}{\text{6}}$ | 5 | 1 | $\frac{\text{1}}{\text{4}}$ | -5 |

**PD** = (Infection rate above 50% – 50%) / (Infection rate above 50%) – (Infection rate below 50%)

= $\frac{\text{66.66}\text{ – 50}}{\text{66.66 – 16.66}}\text{ }$= 0/33

**TCID50** = – [dilution with infection rate above 50% + The logarithmic unit of the dilution factor × (PD)]

= – [–4 + (–1) × (0/33)] = – [–4/33] = +4/33

$\frac{\text{10}^{\text{+}\text{4}\text{/}\text{33}}\text{ TCID50}}{\text{0/08}\text{ml}}\text{ }$= 1/25× 10^6/33^ TCID50

**Table 5. Reed-Muench table for determination of VHSV titer without lentivector inoculation**

| **Percentage of infected wells** | $\frac{\mathbf{A}}{\mathbf{A+B}}$ | **Cumulative frequency of negative wells (B)** | **Cumulative frequency of infected wells (A)** | **number of infected wells** | **log dilution** |
| --- | --- | --- | --- | --- | --- |
| 100/0% | $\frac{\text{16}}{\text{16}}$ | 0 | 16 | $\frac{\text{4}}{\text{4}}$ | -1 |
| 100/0% | $\frac{\text{12}}{\text{12}}$ | 0 | 12 | $\frac{\text{4}}{\text{4}}$ | -2 |
| 88.88/0% | $\frac{\text{8}}{\text{9}}$ | 1 | 8 | $\frac{\text{3}}{\text{4}}$ | -3 |
| 71.42/0% | $\frac{\text{5}}{\text{7}}$ | 2 | 5 | $\frac{\text{3}}{\text{4}}$ | -4 |
| 33.33/0% | $\frac{\text{2}}{\text{6}}$ | 4 | 2 | $\frac{\text{2}}{\text{4}}$ | -5 |

**PD** = (Infection rate above 50% – 50%) / (Infection rate above 50%) – (Infection rate below 50%)

= $\frac{\text{71.42}\text{ – 50}}{\text{71.42 – 33.33}}\text{ }$= 0/56

**TCID50** = – [dilution with infection rate above 50% + The logarithmic unit of the dilution factor × (PD)]

= – [–4 + (–1) × (0/56)] = – [–4/56] = +4/56

$\frac{\text{10}^{\text{+}\text{4}\text{/56}}\text{ TCID50}}{\text{0/08}\text{ml}}\text{ }$= 1/25× 10^6/56^ TCID50

**Table 6. Calculated TCID50 for VHSV in test and control samples**

| **Log TCID_50_ /ml** | **TCID_50_ /ml** | **Sample** |
| --- | --- | --- |
| 2/59 | 1/25× 10^2/50^ | VHSshRNA-1 |
| 2/75 | 1/25× 10^2/66^ | VHSshRNA-2 |
| 2/87 | 1/25× 10^2/77^ | VHSshRNA-3 |
| 6/42 | 1/25× 10^6/33^ | lentivector Scrambled |
| 6/65 | 1/25× 10^6/56^ | Negative vector control |

**Table 7. TCID50 reduction percentage for VHSV in test samples and Scrambled lentivector control**

| **TCID50 reduction percent** | **% SampleTCID_50_ / Scrambled TCID_50_** | **Sample** |
| --- | --- | --- |
| 99/986 | 0/014 | VHSshRNA-1 |
| 99/98 | 0/02 | VHSshRNA-2 |
| 99/973 | 0/027 | VHSshRNA-3 |
| 0 | 100 | lentivector Scrambled |

**Table 8. TCID50 reduction percentage for VHSV in test samples and vector negative control**

| **TCID_50_ reduction percent** | **Negative vector control TCID50/Sample TCID5 %** | **Sample** |
| --- | --- | --- |
| 99/991 | 0/0087 | VHSshRNA-1 |
| 99/99 | 0/01 | VHSshRNA-2 |
| 99/984 | 0/016 | VHSshRNA-3 |
| 0 | 100 | Negative vector control |
